# Application of flexible bronchoscopy in inhalation lung injury

**DOI:** 10.1186/1746-1596-8-174

**Published:** 2013-10-21

**Authors:** Chong Bai, Haidong Huang, Xiaopeng Yao, Shihui Zhu, Bing Li, Jingqing Hang, Wei Zhang, Paul Zarogoulidis, Andreas Gschwendtner, Konstantinos Zarogoulidis, Qiang Li, Michael Simoff

**Affiliations:** 1 Department of Respiratory Medicine, Changhai Affiliated Hospital of the Second Military Medical University, Shanghai 200433, China; 2 Department of Burn, Changhai Affiliated Hospital of the Second Military Medical University, Shanghai 200433, China; 3 Department of Respiratory Medicine, Changzheng Affiliated Hospital of the Second Military Medical University, Shanghai 200003, China; 4 Department of Respiratory Medicine, Shanghai Putuo District People’s Hospital, Shanghai 200060, China; 5 Pulmonary Department, ``G. Papanikolaou´´ General Hopspital, Aristotle University of Thessaloniki, Thessaloniki, Greece; 6 Department of Interventional Pneumology, Ruhrlandklinik, West German Lung Center, University Hospital, University Duisburg-Essen, Essen, Germany; 7 Pathology Department, Hospital of Amberg, Amberg, Germany; 8 Henry Ford Hospital, Pulmonary and Critical Care Medicine, Detroit, MI 48202, USA

**Keywords:** Bronchoscopy, Inhalation, Smoke

## Abstract

**Background:**

As acute inhalational injury is an uncommon presentation to most institutions, a standard approach to its assessment and management, especially using flexible bronchoscopy, has not received significant attention.

**Methods:**

The objective of this study is to evaluate the value of using flexible bronchoscopy as part of the evaluation and management of patients with inhalational lung injury. Twenty-three cases of inhalational lung injury were treated in our three hospitals after a fire in a residential building. The twenty cases that underwent bronchoscopy as part of their management are included in this analysis. After admission, the first bronchoscopy was conducted within 18-72 hours post inhalational injury. G2-level patients were reexamined 24 hours after the first bronchoscopy, while G1-level patients were reexamined 72 hours later. Subsequently, all patients were re-examined every 2-3 days until recovered or until only tunica mucosa bronchi congestion was identified by bronchoscopy.

**Results:**

Twenty patients had airway injury diagnosed by bronchoscopy including burns to the larynx and glottis or large airways. Bronchoscopic classification of the inhalation injury was performed, identifying 12 cases of grade G1 changes and 8 cases of grade G2. The airway injury in the 12 cases of grade G1 patients demonstrated recovery in 2-8 days, in the airway injury of the 8 cases of grade G2 patients had a prolonged recovery with airway injury improving in 6-21 days averaged. The difference in recovery time between the two groups was significant (P <0.05).

**Conclusions:**

The use of flexible bronchoscopy has great value in the diagnosis of inhalational injury without any complications. Its use should be incorporated into clinical practice.

**Virtual slides:**

The virtual slide(s) for this article can be found here: http://www.diagnosticpathology.diagnomx.eu/vs/1476676925108926

## Background

Inhalation injury is a major cause of morbidity and mortality in burn patients. It is, along with age and total burn surface area (TBSA), one of the three most significant predictors of death after thermal injury. The incidence of inhalational injury in burn patients who require hospitalization ranges from 20% to 30%. There is a reported mortality in this same population of 30%. The incidence of respiratory failure is significant after inhalational injuries, with hypoxemia, pneumonia, and respiratory failure with prolonged ventilatory support and extended hospitalizations being common [1-4]. As acute inhalational injury is an uncommon presentation to most institutions, a standard approach to its assessment and management, especially using flexible bronchoscopy, has not received significant attention [5].

On November 15, 2010, a significant fire occurred in a residential building on Jiaozhou Road, in Shanghai, China, which is still known as the "11.15" fire. Fifty-eight people were reported dead and burn patients were sent to a number of hospitals throughout Shanghai. Twenty-three patients were admitted and treated in our three hospitals; bronchoscopy played an important role in the management of twenty of these patients. We report here our experience in the assessment and management of these patients.

## Materials and methods

The study includes all patients who were injured in the fire on Jiaozhou Road, in Shanghai, China on November 15, 2010 and admitted to the following three hospitals: Shanghai Changhai Hospital (20 patients), Shanghai Changzheng Hospital (2 patients) and Shanghai Putuo District People's Hospital (1 case). Excluded were two patients at Changhai Hospital who refused bronchoscopy and a three year old with mild symptoms. The remaining 20 patients had bronchoscopy performed as part of their medical management. The patient population consisted of fourteen males and six females. The patients’ ages ranged from 16-83 years old with an average age of 54.2 ± 8.1 years. Patients had experienced smoke inhalational ranging from 10-120 minutes. Of the twenty patients enrolled, three had grade 2 surface burns, one each of 1%, 4% and 5% total body surface area. In addition one patient had a lower limb crush injury syndrome.

The initial bronchoscopy on all patients was performed within the 18-72 hours of admission to the hospital. An Olympus BF260 videobronchoscope (Olympus Medical Systems Corporation; Tokyo, Japan) was used to perform all airway evaluations. Follow-up airway inspection was conducted according to the patients’ condition.

At the time of the initial bronchoscopic evaluation of the patients, the location of the inhalational injury was described: (a) upper airway injury (burn limited to the larynx and glottis), (b) large airway injury (burn involving the trachea and bronchial tree), or (c) peripheral airway injury (burn involves the terminal bronchioles and/or alveoli) [6].

The visual classification of mucosal airway injury by Chou, et al. was used in the description of bronchoscopic findings of all patients [7]. G0 designates a negative airway examination; G1 mild edema and hyperemia, with or without carbon soot; G2 severe edema and congestion of the airway mucosa, with or without carbon soot; and G3 bronchial ulcers and/or necrosis with the absence of a cough reflex and bronchial secretions. The Gb designation was established for cases that bronchoscopic findings were unclear and mucosal biopsy was performed and found to be positive.

### Statistical analysis

SPSS software was used for statistical analysis. Dose treatment is represented by mean ± standard deviation. Pearson correlation analysis was used and P <0.05 was considered as statistically significant.

### Consent

Written informed consent was obtained from the patient for the publication of this report and any accompanying images.

## Results

### Clinical symptoms

Nineteen of the twenty patients were conscious without breathing difficulties within the first hour after the fire (95%). The single unconscious patient, in addition to inhalational injury, had lower limb crush injury syndrome. Symptoms developed early in the hospital course and included: cough (n = 20, 100%), production of sputum with black carbon soot (n = 18, 90%), hoarseness (n = 16, 80%), dyspnea (n = 10, 50%) and respiratory failure (n = 8, 40%). Physical examination demonstrated, burnt vestibular vibrissa in all twenty patients, and fine moist rales on lung examination in 12 patients (60%). In the eight cases of respiratory failure, one patient was intubated with endotracheal tube, while the remaining seven cases required tracheotomy (See Table 1).

**Table 1:** Symptoms analysis of 20 patients with inhalational injury

	**n=**	**Percentage of total**
Symptoms		
Cough	20	100%
Sputum with black carbon soot	18	90%
Hoarseness	16	80%
Dyspnea	10	50%
Respiratory failure	8	40%
Physical examination		
Burnt vestibular vibrissa	20	100%
Fine moist rales	12	60%

### Identification of site of inhalational injury

Bronchoscopy identified upper and large airway injury patterns in all twenty patients evaluated. Fifteen of the patients (75%) had chest CT findings consistent with pulmonary exudative changes.

### Classification according to the severity of inhalation injury

**Figure 1:**
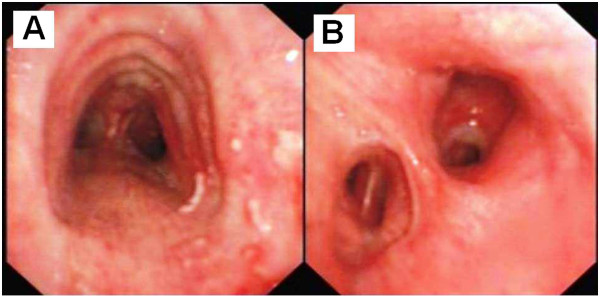
**Bronchoscopic findings in patient A, 18 hours post inhalation injury.** The trachea was classified as grade G1, with mild edema and congestion of the bronchus **(A)**, without carbon soot being identified **(B)**.

**Figure 2:**
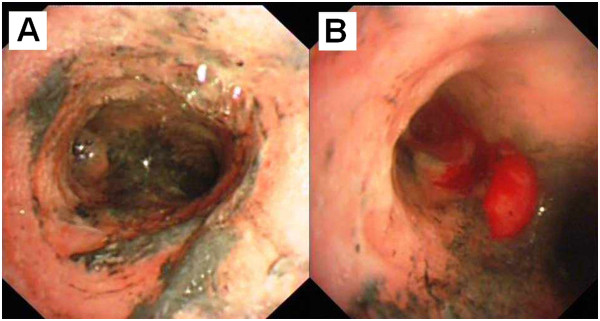
**Bronchoscopic findings of Patient B, eighteen hours after inhalation injury.** The trachea was classified as grade G2 with severe edema and congestion of the bronchus **(A)**. Carbon soot deposition and the formation of pseudomembrane was also demonstrated **(B)**.

**Figure 3:**
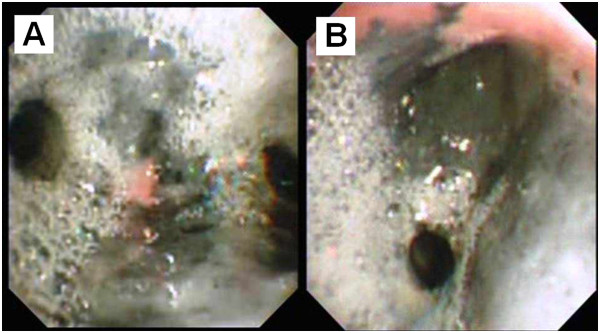
**Bronchoscopic findings of Patient C; A) eighteen hours after inhalation injury. B)** The trachea was classified as grade G2 with severe edema and congestion of the bronchus in addition to carbon soot deposition.

At the time of bronchoscopy, 12 patients (60%) were found to have grade G1airway findings (Figure 1) and eight cases (40%) were graded as G2 airway findings (Figures 2 and 3). The three patients who sustained second degree surface burns (with 1%, 4% and 5% total surface area burned) were found to have grades G1, G2, and G1 respectively at the time of their bronchoscopies. The patient that had sustained lower limb crush injury syndrome was classified as G1. Of the eight patients with respiratory failure, six cases were graded as G1 (75%) and two were graded as G2 (25%).

Table 2 demonstrates the bronchoscopic findings from patient B, eighteen hours post inhalational injury. The Grade G2 findings include severe edema and congestion of the bronchus (Figure 2A) and carbon soot deposition with the formation of a pseudomembrane (Figure 2B). The pseudomembrane was endobronchially resected using biopsy forceps. Mucosal bleeding was noted with the removal of the pseudomembrane (See Table 2).

**Table 2:** **Groups of patients graded according to depth of mucosal damage estimated by fiberoptic bronchoscopy on admission, in line with Chou’s classification**[3]

**Grade**	**Number of patients**	**Findings**
G0	0	Negative (no mucosal damage)
Gb	0	Positive (mucosal damage) confirmed by biopsy
G1	12	Mild edema + hyperemia, with or without carbon soot
G2	8	Severe edema + hyperemia, with or without carbon soot
G3	0	Ulceration, necrosis, no cough reflex or bronchial secretions

### Follow-up

Bronchoscopic re-evaluation of patients for airway injury was standardized as: 72 hours after the initial evaluation in grade G1 patients, repeat bronchoscopy, repeat bronchoscopy 24 hours after the initial evaluation in grade G2 patients. All patients were then followed bronchoscopically every 2-3 days until the tracheal mucosal appearance normalized.

**Figure 4:**
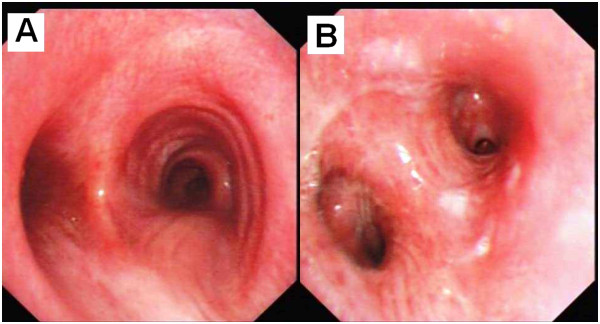
**Bronchoscopic findings in patient C, 11 days post-inhalation injury.** In both the trachea **(A)** and bronchus **(B)** carbon soot and inflammation had resolved with only mild edema and congestion remaining.

To illustrate the healing process, Figure 4 re-examines patient C, eleven days post injury. Initially classified as grade G2 the patient only had mild edema and congestion remaining.

The twelve grade G1 patients had bronchoscopic evidence of recovery in 2-8 days (mean 4.2 ± 1.3 days). The eight grade G2 patients demonstrated bronchoscopic normalization in 6-21 days (mean 15.8 ± 4.2 days). The difference in time to recovery between the two groups was significant (P <0.05). At the follow-up visit (one year) later, there were no obvious bronchoscopic abnormalities in any of the patients (See Table 3).

**Table 3:** Recovery time of 20 patients under bronchoscopy

**Grade**	**Number of patients**	**Recovery time (d)**
G1	12	2-8 (4.2 ± 1.3)
G2	8	6-21 (15.8 ± 4.2)^▲^

^▲^ P < 0.05.

## Discussion

The factors, which most significantly affect the prognosis of patients with burns, are: total body surface area burn, the age of the patient, and the presence of inhalational injury. The reported incidence of inhalational injury burn complications occurs in 7-20% of patients requiring hospitalization [8]. Inhalational injury from burns can increase mortality by 20% and the occurrence of pneumonia by up to 40% [9]. Clinically significant inhalational injuries often do not manifest for three to four days after the exposure [10]. Complications of inhalational injury are not uncommon in patients with burns, coma, or other severe unexplained clinical symptoms [1,11,12]. There are many cases that are easily missed due to inhalation injury that can occur irrespective of burn injuries and clinical manifestations are often not apparent. It is reported that inhalation lung injury can occur in patients with no body surface burn; however, its clinical manifestations are not consistent and can vary from no obvious symptoms to severe respiratory failure [13].

The nineteen patients in our study were conscious at admission, only 15% (three cases) of the patients had body surface burns, and there were no obvious breathing difficulties within the first hour after presentation. Without a high degree of clinical suspicion, the presence of inhalational injury in most of our patients would not have been recognized. By identifying injury early, those patients’ with more advanced exposures can be closely observed, allowing quick clinical response to any change in their medical condition. Particularly in patients without surface burns, the lack of the findings of an early inhalational injury could result in the delay of treatment.

Bronchoscopy is considered the ‘gold standard’ for early evaluation of upper airway injury and can be used to help predict acute lung injury [1,14]. Even when the initial chest examination, chest x-ray, and blood gasses are normal, bronchoscopy can identify large airway injury, a precursor to respiratory complications due to inhalational injury [13,15,16]. One hundred percent of the patients (n = 20) in our study, had bronchoscopic findings of large airway injury, involving both the trachea and proximal bronchial tree. This finding occurred when only 60% of the patients were found to have fine moist rales on chest examination and 75% of patients had computed tomography examinations demonstrating pulmonary exudative changes. Bronchoscopy is therefore a very important tool in the initial evaluation of patients with suspected inhalational lung injury.

Bronchoscopic findings in patients with inhalational injury include: congestion, edema, mucosal ulceration and necrosis. When the inhaled matter contains carbon based soot, the carbon soot will adhere to the mucosal surfaces of all visible airways [13]. Some patients with mild symptoms do not have any visible bronchoscopic findings consistent with inhalational injury. Despite the lack of macroscopic findings, bronchial mucosal biopsies often demonstrate microscopic evidence of inflammatory changes to the airway. When inhalational injury involves the full-thickness of the mucosa as well as the submucosa, the cough reflex as well as mucus secretion production and eventually clearance can become problematic [17]. These patients will have no cough and limited to no secretions when bronchoscopy is performed [10]. In our study, no biopsies were acquired as the treating doctors considered that they would not add to the treatment of the patients. On the contrarary this intervention was considered that it would jeopardize patients health. Classification of inhalational injury by bronchoscopy can be important in the management of patients, despite the fact that no direct link between bronchoscopic grade and mortality has been definitively established.

Gore et al. [13] describes that it is safe to perform flexible bronchoscopy in inhalation lung injury patients, even those with mild airway obstruction. In many cases, it is the personal experience of the clinician that is often the constraining factor for the use bronchoscopy. Those physicians with more experience tend to use bronchoscopy more readily than those who do not. The patients in our study did not demonstrate any clinical problems during our bronchoscopic evaluation of their airways, even those at the G2 level. Bronchoscopy is also of therapeutic value in the management of patients with inhalation lung injury [18,19]. Patients with airway Grade G0 and Gb findings have acute inflammation of the mucous membrane as the main change to the airway, thus there is no bronchoscopic intervention indicated. On the other hand, patients with Grade G3 findings can have necrotic tissue and inflammatory exudates blocking the airway lumen. Immediate intervention with the bronchoscope can be very effective in managing these patients. There are different points of view regarding intervening on the carbon soot findings in patients with Grade G1 and G2; in most instances no intervention is most appropriate. Grade 2 patients can also develop pseudomembranes. In our eight G2 patients, this was an early manifestation of more significant airways disease. We resected the majority of pseudomembranes which were felt to be clinically detrimental to the patients, as complete resection led to more significant mucosal bleeding. Concern always exists that the airways will scar or develop stenosis after an inhalational injury. We identified no significant residual findings at the late bronchoscopic re-examination of the airways, which was consistent with the results reported by Irrazabal et al. [19] From our and reported findings, the recovery time of a bronchial mucosa is longer in Grade G2 injury patients as compared to those with Grade G1 injury. That is, patients with severe inhalation lung injury need a long time for airway mucosal repair, while patients with mild inhalation lung injury need a short time for airway mucosal repair.

In conclusion, the flexible bronchoscope has great value in the diagnosis of inhalation injury without any complications and it should be incorporated into routine clinical practice. The use of flexible bronchoscopy must be done so with the knowledge that the airways will heal themselves if given the appropriate time. The identification of an injury or a pseudomembrane that is not creating a problem for the patient at that time should be left to heal, only using therapeutic procedures for the more significant airways injuries or in patients with respiratory need.

## Abbreviations

TBSA: Total burn surface area.

## Competing interests

The authors declare that they have no competing interests.

## Authors’ contributions

BH and S had full access to all of the data in the study and take responsibility for the integrity of the data and the accuracy of the data analysis. CB: contributed to data acquisition, performed study procedures, interpreted study data, contributed to and reviewed drafts of the manuscript, approved the final version of the manuscript and served as principal author. HH: contributed to data acquisition, performed study procedures, interpreted study data, contributed to and reviewed drafts of the manuscript, approved the final version of the manuscript and served as co-principal author. XY: contributed to data acquisition, performed study procedures, interpreted study data, and writing of manuscript. SZ: contributed to data acquisition, performed study procedures, interpreted study data, and writing of manuscript. BL: contributed to data acquisition, performed study procedures, interpreted study data, and writing of manuscript. JH: contributed to data acquisition, performed study procedures, interpreted study data, and writing of manuscript. WZ: contributed to data acquisition, performed study procedures, interpreted study data, and writing of manuscript. QL: contributed to data acquisition, performed study procedures, interpreted study data, contributed to and reviewed drafts of the manuscript, approved the final version of the manuscript. MS: contributed to interpretation of study data, wrote and reviewed drafts of the manuscript, and approved final version of the manuscript, and served as co-principal author. PZ and KZ contributed in the editing and provided useful insights where necessary. AG assisted in the interpretation of the pathological findings. All authors read and approved the final manuscript.
